# Incidence of thrombotic complications in COVID-19: On behalf of ICODE: The International COVID-19 Thrombosis Biomarkers Colloquium

**DOI:** 10.1007/s11239-021-02475-7

**Published:** 2021-05-28

**Authors:** William J. Jenner, Diana A. Gorog

**Affiliations:** 1 Cardiology Department, East and North Hertfordshire NHS Trust, Stevenage, Hertfordshire UK; 2 Faculty of Medicine, Imperial College, National Heart and Lung Institute, London, UK; 3 School of Life and Medical Sciences, Postgraduate Medical School, University of Hertfordshire, Hatfield, UK

**Keywords:** COVID-19, Thrombosis, Arterial thrombosis, Deep venous thrombosis, Thromboembolism

## Abstract

A high incidence of thrombosis in hospitalised patients with COVID-19 was identified early during the pandemic. Accurately quantifying thrombotic risk may assist prognosis and guide appropriate thromboprophylaxis. Observational studies have estimated the rate of thrombosis in both hospitalised and non-hospitalised patients with COVID-19, and how this corresponds to the severity of illness. In this review, we provide an overview of the incidence and prevalence of arterial and venous thrombotic events in patients with COVID-19 and highlight the limitations in the studies to date. Asymptomatic individuals with COVID-19 and those with mild symptoms are at very low risk of thrombotic complications. However, rates of thrombosis are substantially increased in hospitalised patients, and are strikingly high in those patients who are critically-ill requiring treatment on the intensive care unit and especially those requiring extracorporeal membrane oxygenation. Clinicians managing such patients need to be aware of these risks and take appropriate steps with respect to thromboprophylaxis and heightened clinical vigilance. Large prospective observational studies will more accurately quantify thrombotic rate, and randomized controlled trials are currently investigating optimal thromboprophylactic strategies.

## Highlights

- The rates of arterial and venous thrombotic complications in COVID-19 are low in non-hospitalised individuals with asymptomatic or mild disease.
- Thrombotic risk increases with severity of COVID-19 illness, with those on intensive care being at greatest risk.
- Limitations in the available research include the reliance on retrospective observation studies, variable reporting of types of thrombosis, and the variable use of screening.
- Randomized controlled trials currently underway will guide us as to the optimal thromboprophylaxis strategy to improve outcomes in these patients.

## Introduction

**Fig. 1:**
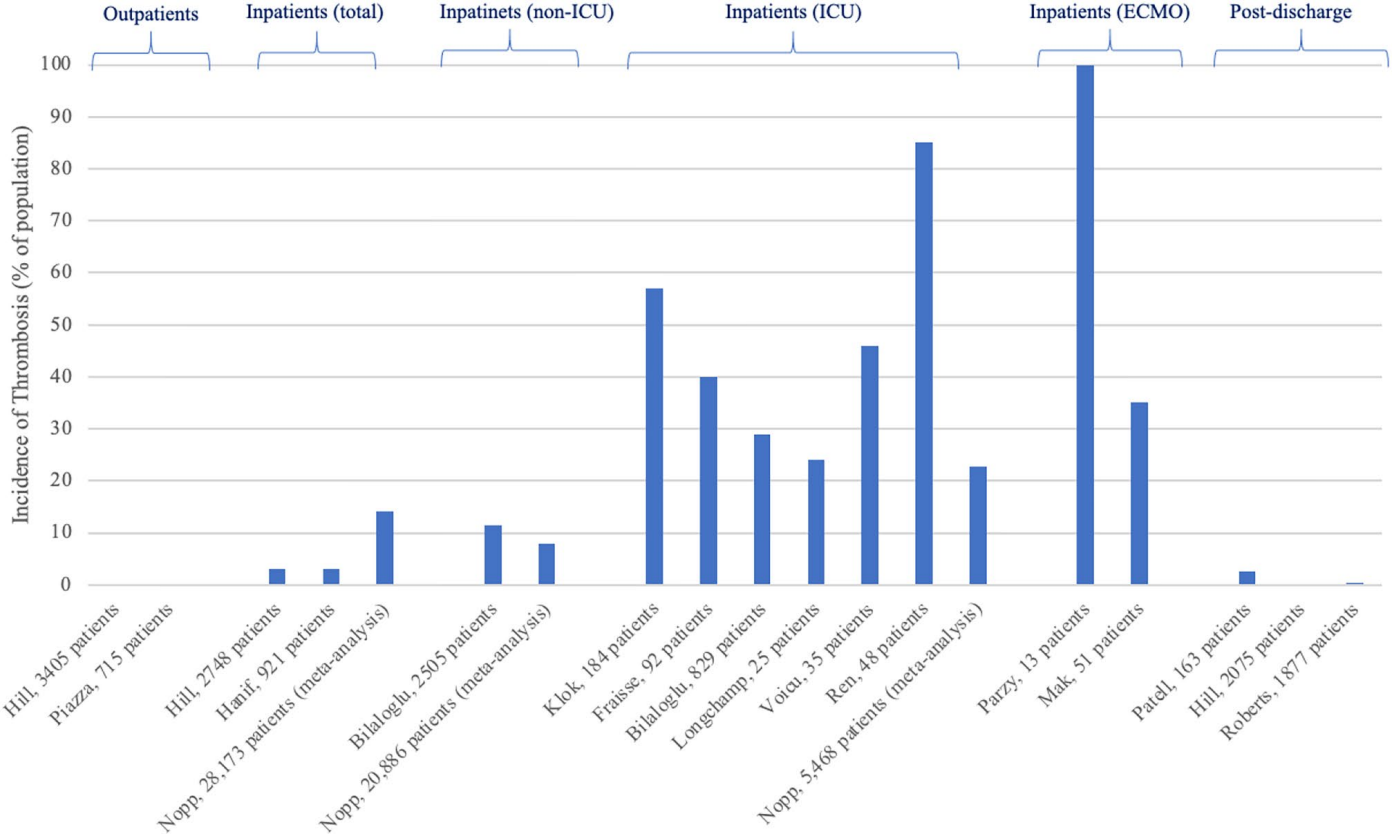
Incidence of thrombotic complications in patients with COVID-19, from selection of described papers

Early in the COVID-19 pandemic, it was identified that the rate of thrombosis in hospitalised patients with COVID-19 was relatively high, and attributed to a pro-thrombotic state [1, 2]. A number of observational studies, typically retrospective cohorts or registries, have been published to document the rates of thrombosis in patients with COVID-19 [3, 4]. However, trying to apply rates of thrombosis between one patient group to another has proven challenging, in particular given that the rate of thrombosis appears to be dependent on the severity of illness, the variability in thresholds used to request imaging and the methods used to identify thrombosis. This article reviews the literature, relating the rates of thrombosis to the severity of COVID-19; namely in those patients managed as an outpatient, inpatient (non-intensive care), ICU, advanced cardiac ICU, and following discharge from hospital (Fig. 1).

## Thrombotic complications of COVID-19 in the community

The incidence of a thrombotic event in patients who test positive for COVID-19 but suffer from either no or mild symptoms or do not require hospitalisation appears to be very low. In a large dataset of 6153 patients by Hill et al., 3405 individuals were identified who had tested positive for COVID-19, but did not require hospitalisation [5]. Searches for thrombotic events, acquired retrospectively from electronic health records, revealed only 3 cases of venous thromboembolism (VTE) recorded in this cohort (0.09%). Piazza et al. performed a retrospective analysis on a cohort of patients who attended a hospital in Boston, USA [6]. Their cohort of 1114 patients with COVID-19 included a non-hospitalized subgroup of 715 patients, in whom no major arterial or venous thrombotic episodes had been recorded in the 30 days subsequent to enrolment, based upon electronic health records. Thrombotic events were reported depending on clinical judgement and subsequent investigations, without any formal screening process. In this subgroup aged 44.8 ± 16.2 years (mean ± SD), COVID-19-related pneumonia was reported in 9.5% of patients, and they had a low frequency of comorbidities, with the most frequent being hypertension (24.5%) and hyperlipidaemia (20.8%).

This suggests that in otherwise well patients who have tested positive for COVID-19 but do not require hospitalisation, the rate of thrombosis is low. However, there are a number of issues, particularly related to the inherent biases of observational studies when the outcomes are likely to be a low frequency. Firstly, patients may not report symptoms, or have treatment for thrombosis managed elsewhere. Secondly, patients may have only had mild symptoms of COVID-19, but then develop a thrombosis that requires hospitalisation, and as such may not be included in the “outpatient” cohorts described above. Finally, publication bias may have contributed to fewer publications that document a low prevalence of thrombotic complications than those reporting unexpectedly high rates of such events.

Nonetheless, there are case reports and series of outpatients who have developed thrombotic events in the context of COVID-19. Uppuluri et al. report the case of a 32-year-old man who developed symptoms of pulmonary embolism (PE) 12 days following an outpatient diagnosis of COVID-19, subsequently confirmed on computed tomography pulmonary angiogram (CTPA) [7]. Akel et al. report a series of six patients who were diagnosed with PE at the point of hospitalisation with COVID-19 [8]. However, four were also described to have evidence of pulmonary opacities consistent with COVID-19 pneumonia, which suggests that these patients may have required hospitalisation even if PE had not been identified.

## Thrombotic complications of Covid-19 amongst hospitalised patients

**Fig. 2:**
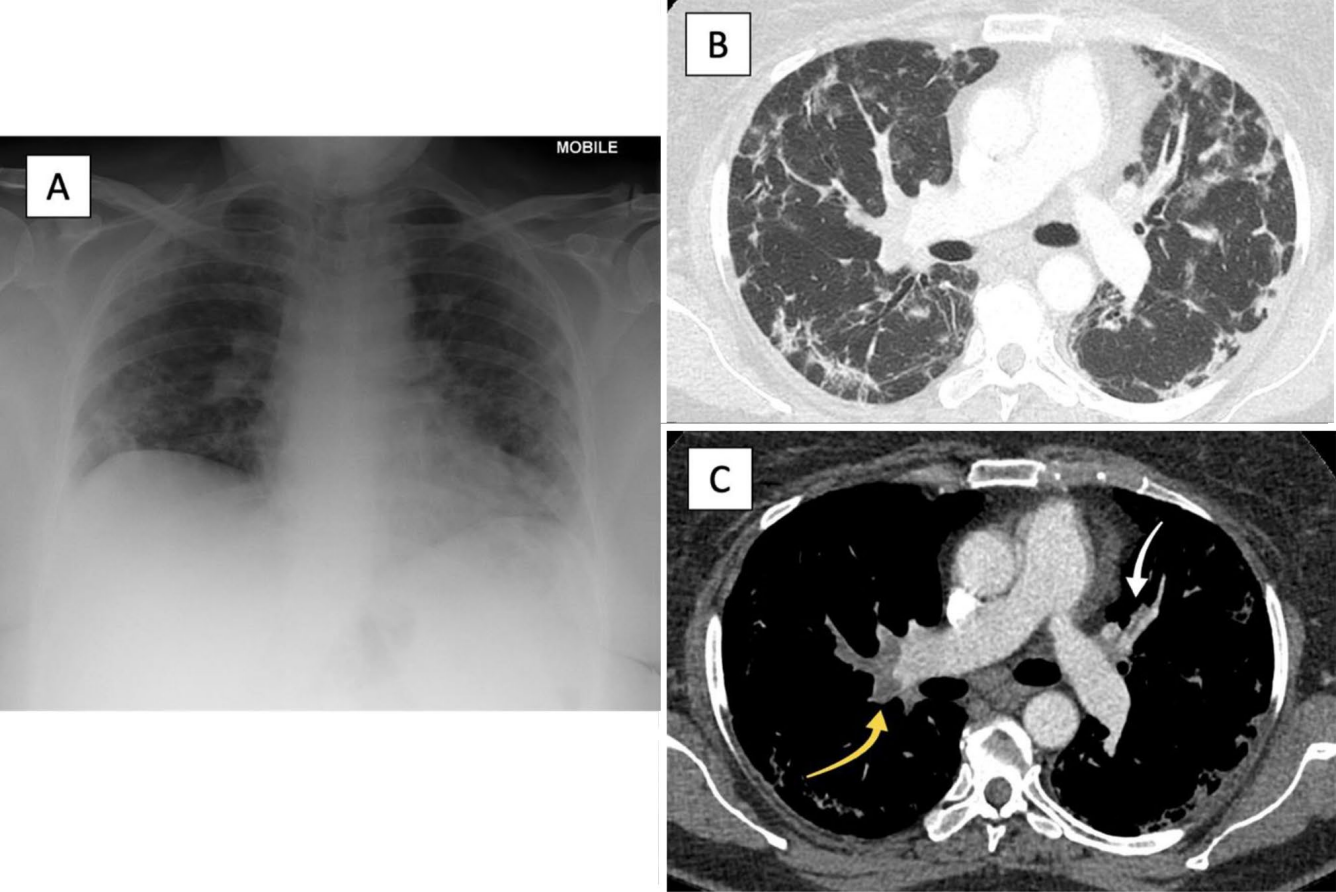
Thoracic imaging of patient with COVID-19 pneumonia with associated pulmonary emboli. Patient managed for COVID-19 pneumonia and pulmonary emboli. Images courtesy of Radiology Department, East and North Hertfordshire NHS Trust. **a** Chest X-ray showing bilateral peripheral consolidation with mid and lower zone predominance, compatible with COVID-19 pneumonia. **b** CTPA lung window showing organising pneumonia pattern typical of COVID-19 pneumonia with peripheral consolidation in a perilobular pattern. **c** CTPA mediastinal window showing large pulmonary embolism within distal right main pulmonary artery (yellow arrow) and small left upper lobe pulmonary embolism (white arrow)

There are much more abundant data regarding the incidence of thrombotic complications in patients who have been hospitalised, with multiple prospective and retrospective studies from populations across the world (Fig. 2). In a large cohort of 2748 patients hospitalised due to COVID-19, 86 patients (3.1%) developed VTE, including deep vein thrombosis (DVT) (1.5%) and PE (1.3%), as well as 7 arterial thromboses (0.3%) [5]. Hanif et al. also report on the rates of thrombosis in hospitalised patients with COVID-19, subdivided into patients who were receiving full anticoagulation prior to admission, and those who received prophylactic anticoagulation [9]. They observed that of 921 patients, 16 patients developed VTE (1.7%), 11 ischaemic strokes (1.2%), and 2 developed limb ischaemia (0.2%). Of the 33 patients who already received anticoagulation prior to admission, none had a thrombotic event. However, 3 (10%) of these anticoagulated patients developed a bleeding event during the hospitalisation, compared to 11 (1.7%) of those patients receiving thromboprophylaxis.

Drawing upon data from multiple databases, comprising 28,173 patients with COVID-19 (94% hospitalised), one meta-analysis estimated an in-hospital prevalence of VTE (PE or DVT) of 14.1% [10]. However, this comprised studies with varying methods of identification, including some that employed routine screening for venous thrombosis. For example, Mazzaccaro et al. performed a screening CTPA on a group of 32 non-ICU hospitalised patients with COVID-19 pneumonia, identifying PE in 21 patients (65.6%) [11]. Alonso-Fernandez et al. carried out a screening CTPA on patients with COVID-19 and an elevated d-dimer > 1 µg/ml, and identified PE in 50% of these patients [12]. It is considered that the rates of VTE in non-screening studies are much lower, estimated at 9.5% (95% CI 7.5–11.7%) by Nopp et al. [10].

Hospitalised patients can be subdivided into those managed on the ward, compared to those managed on intensive care. A recent large meta-analysis comprising 9350 ward (non-ICU) patients across 20 studies identified an overall VTE incidence of 7.1% (95% CI 4.8–9.8) [13]. A further meta-analysis of 20,886 patients in 43 studies reported the incidence of VTE to be 7.9% (95% CI 5.1–11.2), including 3.5% PE (95% CI 2.2–5.1%) and 4.1% DVT (95% CI 2.3–6.4%) [10].

Arterial thromboses, including those in the cerebral, myocardial, mesenteric beds as well as limb ischaemia also warrant consideration, although in general the incidence of arterial thrombotic complications in patients with COVID-19 is less well established than that of VTE. In one large retrospective observational study of 3556 patients, 32 (0.9%) developed an ischaemic stroke, 4 occurring without any symptoms of COVID-19 prior to the event, and in the remainder of cases, stroke developed up to 27 days following COVID-19 symptom onset [14]. Two further reports of COVID-19 in hospital have identified an incidence of arterial thrombosis in 1.8% of 2021 patients, and 5.6% of 531 patients [15, 16].

A meta-analysis of 4466 patients (5 articles) reports a 1.2% pooled incidence of ischaemic stroke in patients with COVID-19 [17]. Incidence of cerebral venous thrombosis with SARS-CoV-2 infection has been estimated at 0.08% [18]. Myocardial injury with associated ST-segment changes on the ECG has also been reported in patients with COVID-19, although how much of this is related to coronary thrombosis remains to be determined [19]. The incidence of myocardial infarction in the hospitalised, non-ICU managed patients with COVID-19 appears low, Piazza et al. reported 1 case in 229 admissions (0.5%) [6].

It currently remains uncertain whether other prothrombotic comorbidities compound the coagulopathy observed with COVID-19. Patell et al. reported thrombosis in COVID-19 across a population of cancer and non-cancer patients in a single centre and identified similar overall incidence of thrombotic events between 45 cancer patients and 353 non-cancer patients [20].

## Thrombotic complications of COVID-19 on the intensive care setting

The incidence of thrombosis in patients with COVID-19 requiring care on the ICU appears to be higher than that in non-ICU hospitalised patients. Klok et al. reported on 184 ICU-treated patients with COVID-19, and based on clinical suspicion, identified a crude cumulative thrombosis incidence of 57% (95% CI 47–67%), comprising 65 patients with a PE, 3 with other venous thromboses, and 7 patients with arterial thromboses [1, 21]. Fraisse et al. similarly identified a high rate of thrombosis, with a 40% rate of thrombosis in 92 patients [22].

In one of the largest cohort studies, Bilaloglu et al. reported on 829 ICU patients with COVID-19, where thrombosis diagnosis was made based on clinical suspicion [23]. They found an overall 29% incidence of thrombosis, comprising of PE (6.2%), DVT (9.4%), stroke (3.7%) and myocardial infarction (13.9%), with an overall mortality rate of 54%. This compares to an overall incidence of thrombosis of 11.5% in 2505 non-ICU hospitalised patients, comprising PE (2.2%), DVT (2.0%), stroke (0.9%) and myocardial infarction (7.3%). In a meta-analysis of 2928 patients on ICU with COVID-19, our team identified a cumulative thrombotic incidence of 34% [24].

The incidence of thrombosis is particularly high in the ICU patients when systematic screening with ultrasonography is performed to identify DVT. Longchamp et al. performed lower limb venous ultrasound in 25 ICU patients, confirming DVT in 6 patients (24%) [25]. Another study in which lower limb venous ultrasound was routinely performed in 56 ICU patients, identified DVT in 26 patients (46%) [26]. Ren et al. screened 48 ICU patients and identified DVT in 41 patients (85%) [27].

## Thrombotic complications of COVID-19 in patients on ECMO

A proportion of the most critically unwell patients with COVID-19 require advanced cardiac ICU support including extracorporeal membrane oxygenation (ECMO). Patients requiring ECMO require anticoagulation to prevent circuit thrombosis, and are known to exhibit high rates of both thrombotic and bleeding complications [28]. Several studies have published the incidence of thrombotic complication in patients with COVID-19 on ECMO, and noted particularly high rates of thrombosis, often despite therapeutic anticoagulation. A small cohort study in which CT scans were performed in all patients requiring admission for veno-venous ECMO, reported VTE in all 13 patients, with 3 (23%) developing significant bleeding [29]. These patients were compared with a similar group of patients previously admitted with influenza (n = 10), and bacterial community acquired pneumonia (n = 24), and although the rate of VTE was higher in the COVID-19 group, this was not statistically significant. A subset of another ICU cohort study comprised 35 patients on ECMO, and identified at least one episode of thrombosis in 22 patients (63%) [30]. Mak et al. performed a CTPA in 51 patients with COVID-19 and identified pulmonary filling defects in 18 (35.3%), with 28 (49%) patients demonstrating wedge-shaped low-attenuation areas suggestive of lung ischaemia [31].

The added thrombotic complications related to ECMO include that of the extra-corporeal system: central lines, the centrifugal pump and the oxygenator. The presence of thrombus in the extra-corporeal system can cause complete system failure with a high risk of mortality. Beyls et al. describe one such event, where a patient with COVID-19 died of a major oxygenator thrombosis shortly after starting veno-venous ECMO [32].

Bemtgen et al. describe a high incidence of circuit thrombosis in COVID-19 patients, despite continuous intravenous heparin infusion, adjusted to an APTT of 40–50 s [33]. Comparing small groups of patients with COVID-19 requiring ECMO to those on ECMO without COVID-19, has revealed a higher prevalence of thrombotic complications in those with COVID-19. In 11 patients with COVID-19, there were 9 pump changes, and 4 system changes, representing a significantly higher rate of thrombotic complications in COVID-19 patients (64%), compared to non-COVID-19 patients (18%) [33]. A large worldwide registry of patients receiving ECMO for COVID-19 has documented the rates of circuit change at 15%, membrane lung failure in 8%, cannula problems in 6%, and pump failure in 0.8%, although it cannot be determined what proportion of these are directly attributable to thrombosis [34].

## Thrombotic complications of COVID-19 following discharge from hospital

The risk of thrombosis is highest in patients with severe COVID-19, but the risk of thromboembolism does not cease at hospital discharge. Given that some patients will have ongoing symptoms for weeks following discharge, and during this time may be at a lower functional state with reduced mobility than prior to the illness, and given the potential ongoing, albeit reduced, inflammatory state post-discharge, there remains a risk of increased tendency to thrombosis. To date, there are limited studies investigating the risk of VTE in patients following hospital discharge. Patell et al. reviewed 163 patients with COVID-19 followed for a median 30 (IQR 17–46) days following hospital discharge [35]. Patients were excluded if they were discharged on prophylactic or therapeutic anticoagulation. During follow-up, there was a cumulative incidence of 2.5% (95% CI 0.8 to 7.6) of thrombotic events, with VTE alone occurring in 0.6% (95% CI 0.1–4.6) and 3.7% (95% CI 1.4–9.8) incidence of haemorrhage.

Another retrospective analysis compared a large cohort of 1877 patients discharged following admission with COVID-19, with a cohort of 18,159 patients discharged from 2019 following other medical admissions [36]. Amongst patients with COVID-19, a rate of 4.8 VTEs per 1000 patients discharged was reported, which was similar to that reported in patients discharged the year prior with alternative medical diagnoses (3.1 VTEs per 1000 patients, p = 0.2). Finally, Hill et al. observed 3 post discharge VTEs in 2075 patients (incidence 0.14%) [5].

## Discussion

Thrombotic complications, in particular venous thrombosis and thromboembolism are highly prevalent in patients with COVID-19. From the studies available, it seems evident that as the severity of COVID-19 illness increases, requiring higher levels of care, so does the rate of thrombosis.

Several guidelines have been produced, in part building upon these data to provide advice to clinicians as how to interpret this when implementing thromboprophylaxis strategy [37–39]. However, there are differences in the guidelines, likely a product of the limitations of the data available, and lack of prospective interventional studies to guide optimal treatment [40].

There are a number of limitations pertaining to the data reported here on the prevalence of thrombotic complications. The vast majority of studies are retrospective observational studies. Secondly, identification of thrombosis is generally dependent on the clinical suspicion, any conventional clinical features of VTE are frequently absent or difficult to recognise especially in those on the ICU. There is also the logistical challenge of performing imaging tests in patients that may expose a technician to COVID-19, as well as the challenge of transporting critically unwell patients to CT scanners. Amongst individuals with COVID-19 in the community, who are either not admitted or discharged home without admission, VTE may be missed and under-reported.

In patients with critical illness with COVID-19, especially towards the end of life, when identification of thrombosis may not trigger change in management, VTE may not be investigated with confirmatory imaging. Under-reporting of VTE in critically-ill patients is supported by autopsy data, with one autopsy study identifying DVT in 7 of 12 patients (58%) with PE being the direct cause of death in 4 patients (33%) [41].

Another important limitation is the variable reporting of different types of thrombosis in different studies. Several studies only report DVT, whilst others only PE, and few reports on arterial thrombosis. Furthermore, given that the vast majority of studies in ICU-treated patients indicate that standard anticoagulant thromboprophylaxis may be insufficient, bleeding events have been documented in very few studies to date. A recent large meta-analysis of thrombotic complications in patients with COVID-19 identified just 5 articles documenting bleeding events, compared to 44 documenting thrombotic events [13].

Lastly, studies employing routine screening for VTE report a higher incidence of thrombotic complications than those relying on clinical suspicion. However, whether identification and subsequent treatment of small and potentially asymptomatic thrombotic complications, for example subsegmental PEs, will improve outcome, is unclear and as such, the role of routine screening remains debatable [42].

This paper demonstrates high rates of thrombosis in patients with severe illness related to COVID-19. An important hypothesis being considered is whether treating patients who are at the highest risk of thrombosis with a higher dose of prophylactic anticoagulation will reduce the thrombotic burden and improve outcomes. One of the first randomized controlled trials to report on this hypothesis is the INSPIRATION trial, which randomised 562 ICU inpatients to either an intermediate or standard dose of prophylactic anticoagulation [43]. There was no significant difference between the two arms in terms of the primary outcome: a composite of thrombosis, treatment with ECMO and all-cause mortality. There was a statistically significant higher incidence of severe thrombocytopenia, and a tendency towards higher rates of bleeding in the intermediate dose arm; complications that may limit the role of anticoagulation in reducing the thrombotic burden. One observation from this trial was that the incidence of venous thrombosis in the control arm, at 3.5%, was substantially lower that identified in the previously described meta-analysis, and requires further assessment in prospective studies [24].

## Conclusion

The association of COVID-19 with venous thrombotic complications is undisputed, with the severity of COVID-19 being directly proportional to the risk of thrombotic complications. The prevalence of arterial thrombotic events is still not well understood. Large prospective observational studies are urgently needed to better define thrombosis risk, in varying cohorts including those with advancing age, diabetes, obesity and in Black, Asian and minority ethnic cohorts, including in the outpatient and post-discharge period.

## Abbreviations

CTPA: Computed tomography pulmonary angiogram
COVID-19: Coronavirus disease 2019
DVT: Deep venous thrombosis
ECMO: Extra-corporeal membrane oxygenation
ICU: Intensive care unit
PE: Pulmonary embolism
VTE: Venous thromboembolism

## References

[1] Klok FA, Kruip MJHA, van der Meer NJM (2020). Incidence of thrombotic complications in critically ill ICU patients with COVID-19. Thromb Res.

[2] Cui S, Chen S, Li X (2020). Prevalence of venous thromboembolism in patients with severe novel coronavirus pneumonia. J Thromb Haemost.

[3] Middeldorp S, Coppens M, van Haaps TF (2020). Incidence of venous thromboembolism in hospitalized patients with COVID-19. J Thromb Haemost.

[4] Helms J, Tacquard C, Severac F (2020). High risk of thrombosis in patients with severe SARS-CoV-2 infection: a multicenter prospective cohort study. Intensive Care Med.

[5] Hill JB, Garcia D, Crowther M (2020). Frequency of venous thromboembolism in 6513 patients with COVID-19: a retrospective study. Blood Adv.

[6] Piazza G, Campia U, Hurwitz S (2020). Registry of arterial and venous thromboembolic complications in patients with COVID-19. J Am Coll Cardiol.

[7] Uppuluri EM, Shapiro NL (2020). Development of pulmonary embolism in a nonhospitalized patient with COVID-19 who did not receive venous thromboembolism prophylaxis. Am J Health Syst Pharm.

[8] Akel T, Qaqa F, Abuarqoub A, Shamoon F (2020). Pulmonary embolism: a complication of COVID 19 infection. Thromb Res.

[9] Hanif A, Khan S, Mantri N (2020). Thrombotic complications and anticoagulation in COVID-19 pneumonia: a New York City hospital experience. Ann Hematol.

[10] Nopp S, Moik F, Jilma B (2020). Risk of venous thromboembolism in patients with COVID-19: a systematic review and meta-analysis. Res Pract Thromb Haemost.

[11] Mazzaccaro D, Giacomazzi F, Giannetta M (2020). Non-overt coagulopathy in non-ICU patients with mild to moderate COVID-19 pneumonia. J Clin Med.

[12] Alonso-Fernández A, Toledo-Pons N, Cosío BG (2020). Prevalence of pulmonary embolism in patients with COVID-19 pneumonia and high D-dimer values: a prospective study. PLoS ONE.

[13] Jiménez D, García-Sanchez A, Rali P (2020). Incidence of VTE and bleeding among hospitalized patients with coronavirus disease 2019. Chest.

[14] Yaghi S, Ishida K, Torres J (2020). SARS-CoV-2 and stroke in a New York Healthcare System. Stroke.

[15] Rey JR, Caro-Codón J, Poveda Pineda D (2020). Arterial thrombotic complications in hospitalized patients with COVID-19. Rev Esp Cardiol.

[16] Fournier M, Faille D, Dossier A (2021). Arterial thrombotic events in adult inpatients with COVID-19. Mayo Clin Proc.

[17] Tan Y-K, Goh C, Leow AST (2020). COVID-19 and ischemic stroke: a systematic review and meta-summary of the literature. J Thromb Thrombolysis.

[18] Baldini T, Asioli GM, Romoli M (2021). Cerebral venous thrombosis and severe acute respiratory syndrome coronavirus-2 infection: a systematic review and meta-analysis. Eur J Neurol.

[19] Bangalore S, Sharma A, Slotwiner A (2020). ST-segment elevation in patients with Covid-19—a case series. N Engl J Med.

[20] Patell R, Bogue T, Bindal P (2020). Incidence of thrombosis and hemorrhage in hospitalized cancer patients with COVID-19. J Thromb Haemost.

[21] Klok FA, Kruip MJHA, van der Meer NJM (2020). Confirmation of the high cumulative incidence of thrombotic complications in critically ill ICU patients with COVID-19: an updated analysis. Thromb Res.

[22] Fraissé M, Logre E, Pajot O (2020). Thrombotic and hemorrhagic events in critically ill COVID-19 patients: a French monocenter retrospective study. Crit Care.

[23] Bilaloglu S, Aphinyanaphongs Y, Jones S (2020). Thrombosis in hospitalized patients with COVID-19 in a New York City Health System. JAMA.

[24] Jenner WJ, Kanji R, Mirsadraee S (2021). Thrombotic complications in 2928 patients with COVID-19 treated in intensive care: a systematic review. J Thromb Thrombolysis.

[25] Longchamp A, Longchamp J, Manzocchi-Besson S (2020). Venous thromboembolism in critically ill patients with Covid-19: results of a screening study for deep vein thrombosis. Res Pract Thromb Haemost.

[26] Voicu S, Bonnin P, Stépanian A (2020). High prevalence of deep vein thrombosis in mechanically ventilated COVID-19 patients. J Am Coll Cardiol.

[27] Bin Ren, Feifei Yan, Zhouming Deng (2020). Extremely high incidence of lower extremity deep venous thrombosis in 48 patients with severe COVID-19 in Wuhan. Circulation.

[28] Sklar MC, Sy E, Lequier L (2016). Anticoagulation practices during venovenous extracorporeal membrane oxygenation for respiratory failure. A systematic review. Ann ATS.

[29] Parzy G, Daviet F, Puech B (2020). Venous thromboembolism events following venovenous extracorporeal membrane oxygenation for severe acute respiratory syndrome coronavirus 2 based on CT scans. Crit Care Med.

[30] Mirsadraee S, Gorog DA, Mahon CF (2021). Prevalence of thrombotic complications in ICU-treated patients with coronavirus disease 2019 detected with systematic CT scanning. Crit Care Med.

[31] Mak SM, Mak D, Hodson D (2020). Pulmonary ischaemia without pulmonary arterial thrombus in COVID-19 patients receiving extracorporeal membrane oxygenation: a cohort study. Clin Radiol.

[32] Beyls C, Huette P, Abou-Arab O (2020). Extracorporeal membrane oxygenation for COVID-19-associated severe acute respiratory distress syndrome and risk of thrombosis. Br J Anaesth.

[33] Bemtgen X, Zotzmann V, Benk C (2020). Thrombotic circuit complications during venovenous extracorporeal membrane oxygenation in COVID-19. J Thromb Thrombolysis.

[34] Barbaro RP, MacLaren G, Boonstra PS (2020). Extracorporeal membrane oxygenation support in COVID-19: an international cohort study of the Extracorporeal Life Support Organization registry. The Lancet.

[35] Patell R, Bogue T, Koshy A (2020). Postdischarge thrombosis and hemorrhage in patients with COVID-19. Blood.

[36] Roberts LN, Whyte MB, Georgiou L (2020). Postdischarge venous thromboembolism following hospital admission with COVID-19. Blood.

[37] Thachil J, Tang N, Gando S (2020). ISTH interim guidance on recognition and management of coagulopathy in COVID-19. J Thromb Haemost.

[38] Barnes GD, Burnett A, Allen A (2020). Thromboembolism and anticoagulant therapy during the COVID-19 pandemic: interim clinical guidance from the anticoagulation forum. J Thromb Thrombolysis.

[39] Spyropoulos AC, Levy JH, Ageno W (2020). Scientific and Standardization Committee communication: clinical guidance on the diagnosis, prevention, and treatment of venous thromboembolism in hospitalized patients with COVID-19. J Thromb Haemost.

[40] Flaczyk A, Rosovsky RP, Reed CT (2020). Comparison of published guidelines for management of coagulopathy and thrombosis in critically ill patients with COVID 19: implications for clinical practice and future investigations. Crit Care.

[41] Wichmann D, Sperhake J-P, Lütgehetmann M (2020). Autopsy findings and venous thromboembolism in patients with COVID-19: a prospective cohort study. Ann Intern Med.

[42] Minet C, Potton L, Bonadona A (2015). Venous thromboembolism in the ICU: main characteristics, diagnosis and thromboprophylaxis. Crit Care.

[43] Sadeghipour P, Talasaz AH, INSPIRATION Investigators (2021). Effect of intermediate-dose vs standard-dose prophylactic anticoagulation on thrombotic events, extracorporeal membrane oxygenation treatment, or mortality among patients with COVID-19 admitted to the intensive care unit: the INSPIRATION Randomized Clinical Trial. JAMA.

